# Metal–ligand pair anisotropy in a series of mononuclear Er–COT complexes†
†Electronic supplementary information (ESI) available. CCDC 1831312–1831315. For ESI and crystallographic data in CIF or other electronic format see DOI: 10.1039/c8sc01361f


**DOI:** 10.1039/c8sc01361f

**Published:** 2018-07-31

**Authors:** J. D. Hilgar, M. G. Bernbeck, B. S. Flores, J. D. Rinehart

**Affiliations:** a Department of Chemistry and Biochemistry , University of California – San Diego , La Jolla , CA 92093 , USA . Email: jrinehart@ucsd.edu

## Abstract

Synthetic control of the crystal field has elevated lanthanides to the forefront of single-molecule magnet (SMM) research, yet the resultant strong, predictable single-ion anisotropy has thus far not translated into equally impressive molecule-based magnets of higher dimensionality. This roadblock arises from the dual demands made of the crystal field: generate anisotropy and facilitate magnetic coupling. Here we demonstrate that particular metal–ligand pairs can dominate the single-ion electronic structure so fully that the remaining coordination sphere plays a minimal role in the magnitude and orientation of the magnetic anisotropy. This Metal–Ligand Pair Anisotropy (MLPA) effectively separates the crystal field into discrete components dedicated to anisotropy and magnetic coupling. To demonstrate an MLPA building unit, we synthesized four new mononuclear complexes that challenge the electronic structure of the iconic lanthanocene ([Ln(COT)_2_]^+^; COT^2–^ = cyclooctatetraene dianion) complex which is known to generate strong anisotropy with Ln = Er^3+^. Variation in symmetry and coordination strength for Er(COT)I(THF)_2_ (THF = tetrahydrofuran) (**1**), Er(COT)I(Py)_2_ (Py = pyridine) (**2**), Er(COT)I(MeCN)_2_ (MeCN = acetonitrile) (**3**), and Er(COT)(Tp*) (Tp* = tris(3,5-dimethyl-1-pyrazolyl)borate) (**4**) shows that the Er–COT unit stabilizes anisotropy despite deliberate de-optimization. All four half-sandwich complexes display SMM behavior with effective energy barriers of *U*_eff_ = 95.6(9), 102.9(3.1), 107.1(1.3), and 133.6(2.2) cm^–1^ for **1–4** by a multi-relaxation-process fitting. More importantly, the basic state splittings remain intact and the anisotropy axes are within several degrees of normal to the COT^2–^ ring according to complete active space self-consistent field (CASSCF) calculations. Further investigation of the MLPA conceptual framework is warranted as it can provide building units with well-defined magnetic orientation and strength. We envision that the through-barrier processes observed herein, such as quantum tunneling, can be mitigated by formation of larger clusters and molecule-based materials.

## Abstract 2

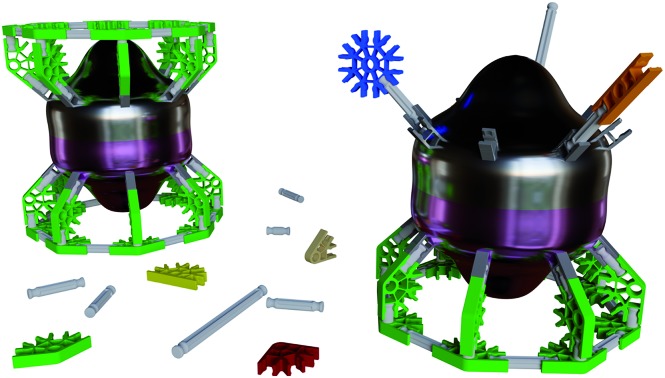
A series of mononuclear erbium complexes demonstrates a pathway to preserving anisotropy for their use as magnetic building blocks.

## Introduction

A strongly interdisciplinary research field has formed around the discovery that quantized states of discrete molecules can lead to superparamagnetism. This field, known as single-molecule magnetism (SMM), aims to understand, control, and optimize the molecular coordination chemistry that determines these magnetic properties. Since SMM research began, there have been proposals for how it could be applied technologically.^1^ These include the construction of devices with spin-dependent transport modulated through single molecules^2^ and using SMMs as building blocks for the construction of higher dimensionality magnetic materials.^3^ This second proposal is intriguing because molecular synthesis techniques have been shown to allow custom design of near-perfect single-ion anisotropy^4^ – a feat unattained in even the strongest known solid state magnetic materials. For this single-ion anisotropy to translate into a strongly coercive molecule-based magnet, however, it must be coupled to a large magnetization. This leads to a fundamental challenge in bottom-up approaches to magnetic materials: facilitating exchange coupling interactions between magnetic centers involves a strong perturbation of the ligand field through introduction of a bridging ligand.^5^ Therefore, the more precisely the single-ion anisotropy is controlled, the more difficulty there is preserving it through the dimensional expansion process to multinuclear clusters and networks with 1-3-dimensional connectivity. Conversely, in a strongly coupled material, it is difficult to introduce anisotropy because the ligand field symmetry is already largely set.

Emphasis on either anisotropy or magnetic coupling individually has led to an accelerating series of advances, with SMM anisotropy barriers (*U*_eff_) rising by over an order of magnitude,^6^ and magnetic coupling strengths allowing collective spin behavior to be observed at much higher temperatures.^7^ These advancements have been facilitated by intuitive models that inform metal and ligand choice, allowing synthetic chemists to focus on specific, optimized targets.7c,^8^ There are, however, a dearth of simple models for the synthesis of robust-anisotropy building units. Ideally, such a building unit would preserve both the magnitude and orientation of the anisotropy axis in a single-ion SMM independent of further structural expansion. This fundamental unit of anisotropy would necessarily involve a single spin center and at least one ligand to anchor the anisotropy axis without saturating the coordination sphere. We refer to this design principle as Metal Ligand Pair Anisotropy (MLPA).

**Fig. 1:**
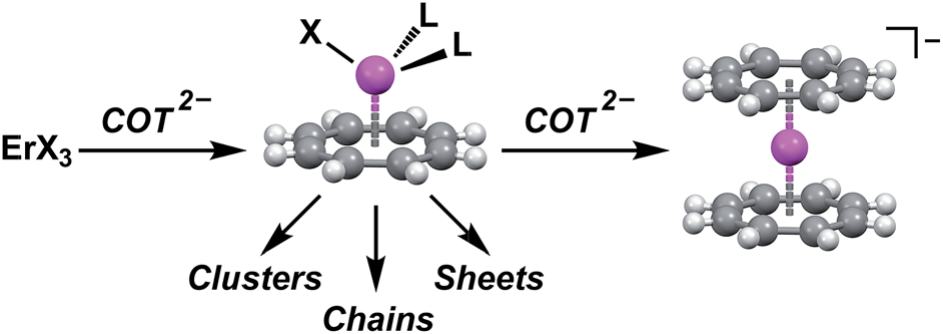
General scheme for the preparation of Er–COT-based single-molecule magnets from an erbium trihalide. Introducing 1 equivalent of COT^2–^ yields a coordinatively-reactive half-sandwich complex that can serve as an anisotropic synthon for clusters or coordination polymers. Introducing an additional equivalent of COT^2–^ yields the magnetically stronger, but coordinatively inert, Er(COT)_2_^–^ fragment.

**Fig. 2:**
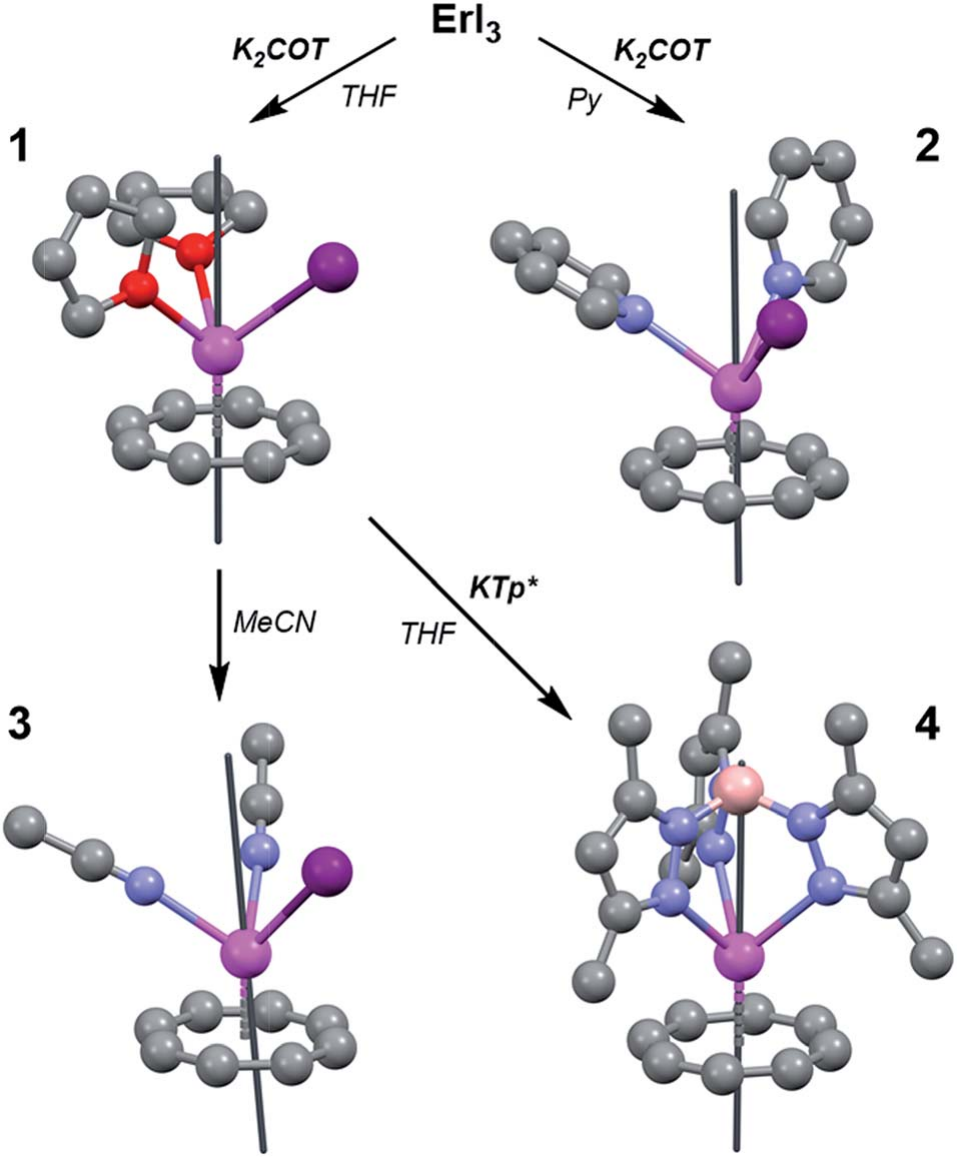
Solid-state structures of **1–4** with spheres representing Er (pink), I (purple), O (red), N (blue), C (gray), and B (salmon). Hydrogen atoms and outer-sphere solvent have been omitted for clarity. Black lines depict the direction of the main magnetic axis of the ground Kramers doublet.

Herein we demonstrate the validity of MLPA using the combination of the high magnetic moment, strongly spin–orbit coupled Er^3+^ cation and the dianionic cyclooctatetraene ligand (COT^2–^) (Fig. 1). We chose the Er–COT motif because the non-axial ligation of a hoop-like COT^2–^ provides considerable stabilization of prolate, high-moment, crystal field states on Er^3+^ without saturating the coordination sphere. Indeed, the prospects of this magnetic unit are bolstered by several examples of high-anisotropy sandwich complexes containing Er^3+^ and COT^2–^,^9^ and a dinuclear complex, [Er(μ_2_-Cl)(COT)(THF)]_2_.^10^ To rigorously demonstrate that MLPA is preserved in the Er–COT unit, we have synthesized and characterized a series of new mononuclear Er–COT complexes: Er(COT)I(THF)_2_ (THF = tetrahydrofuran) (**1**), Er(COT)I(Py)_2_ (Py = pyridine) (**2**), Er(COT)I(MeCN)_2_ (MeCN = acetonitrile) (**3**), and Er(COT)(Tp*) (Tp* = tris(3,5-dimethyl-1-pyrazolyl)borate) (**4**) (Fig. 2). Each of these complexes represents an electronic perturbation of the Er–COT unit designed to test its resistance to anisotropy lowering. Additionally, compounds **1–3** possess a coordinatively-reactive hemisphere opposite the COT ring which will lend them well to use as building units for multinuclear systems and higher dimensional magnets.

## Results and discussion

### Synthesis and structural characterization

Complexes **1–4** were synthesized *via* air- and water-free techniques (Fig. 2). Briefly, **1** is formed from the addition of one equivalent of K_2_COT to a stirring suspension of anhydrous ErI_3_ in THF at –45 °C. The coordinated THF in this complex was found to be quite labile and **3** could be formed by dissolving **1** in MeCN at 50 °C. The pyridine adduct, **2**, can also be prepared *via* solvent exchange, but higher yields are obtained from the direct reaction of ErI_3_ and K_2_COT in pyridine at –45 °C. Reaction of **1** with the scorpionate ligand salt KTp* in THF gave the metathesis product **4**. Given the relative ease with which **1** undergoes solvent and halide exchange, we anticipate that it will find use as an MLPA building unit.

Solid-state structures of the Er–COT compounds **1–4** were determined by single crystal X-ray crystallographic methods using a Mo K(α) source (Fig. 2). The Er–COT (centroid) distances of **1–3** are quite similar *d*_avg_ = 1.763(12) Å. Analogs of **1** have been reported for five other lanthanides (Ln(COT)I(THF)_*x*_, *x* = 3: La,^11^ Ce,^12^ Nd,^13^ Sm;^11^*x* = 2: Tm^14^). Compound **4** contains an Er–COT fragment chelated by the tridentate Tp*^–^ anion with an additional equivalent of THF in the crystal lattice. Elemental analyses and magnetic studies indicate that this THF is removed *in vacuo*. The Er–COT (centroid) distance in **4** is longer than **1–3** (1.836 Å), presumably a result of the steric bulk of the Tp*^–^ ligand that points toward the COT^2–^ ring. Importantly, no crystallographic symmetry is enforced at the erbium center in these complexes and they offer a variety of ligand strengths and orientations. We thus conclude that **1–4** are good test cases for the preservation of MLPA with the Er–COT motif.

### Static magnetic properties

Zero-field cooled dc magnetic susceptibilities for each compound were measured between 2 and 300 K under a 1000 Oe applied field (Fig. S5–S12, ESI†). At 300 K, experimental *χ*_M_*T* values (*χ*_M_*T* = 11.63, 11.29, 11.59, 12.25 cm^3^ K mol^–1^ for **1**, **2**, **3**, and **4**, respectively) are in good agreement to that expected for an Er^3+^ ensemble with equal population of the ground *J* = 15/2 manifold (*χ*_M_*T*_theory_ = 11.48 cm^3^ K mol^–1^, *g* = 6/5). Upon cooling, **1–4** exhibit a monotonic decline in *χ*_M_*T* as their crystal field-split m_*J*_ manifold depopulates. Furthermore, all compounds show a notable drop in *χ*_M_*T* below *ca.* 5 K which can be indicative of SMM behavior on the time-scale of the dc scan.

Isothermal magnetization studies were conducted between 2 and 300 K at maximum external fields of ±7 T (Fig. S5–S12, ESI†). All compounds display typical saturation behavior as the external field is swept from 0 to 7 T and saturate with similar molar magnetizations (*M*_Sat_ = 4.92, 4.62, 4.22, 4.58 *μ*_B_ mol^–1^ for **1–4**, respectively). As the field is swept back from 7 to 0 T, magnetic hysteresis is observed in each complex. As expected for asymmetric mononuclear complexes, there is no remnant magnetization (*M*_R_ = 0 *μ*_B_ mol^–1^) on the timescale of our magnetization experiments. This ‘butterfly’ hysteretic behavior is common to SMMs relaxing *via* a quantum tunneling of the magnetization (QTM) pathway.

### Dynamic magnetic properties

**Fig. 3:**
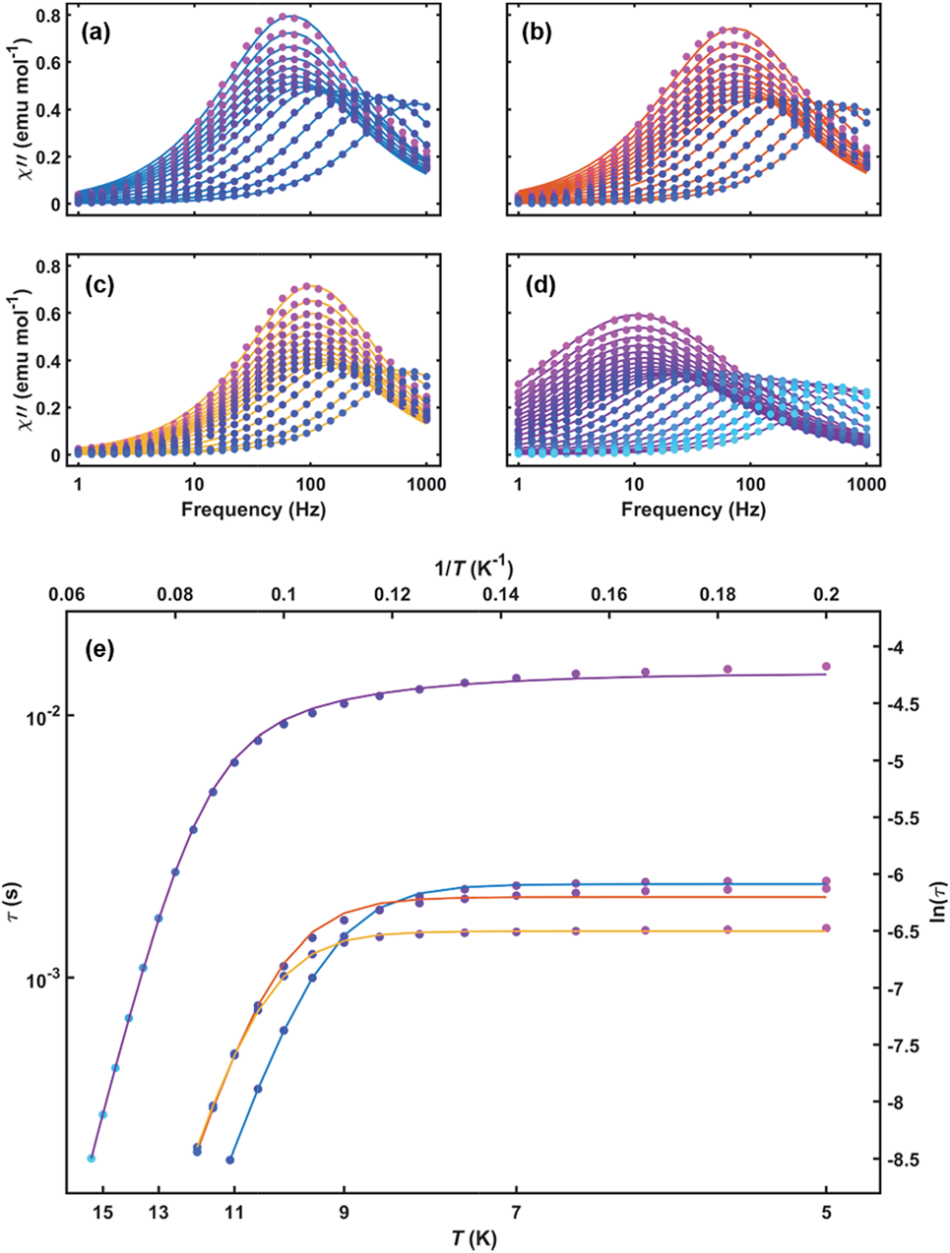
Out-of-phase susceptibility *versus* frequency for (a) **1**, (b) **2**, (c) **3** and (d) **4** with both measured data (circles) and Debye model fits (lines). (e) Arrhenius plot of relaxation time *versus* temperature for **1–4**. Solid lines are fits to eqn (1) (**1–3**) and eqn (2) (**4**) over all measured temperatures. Line colors: **1** (blue), **2** (red), **3** (orange), and **4** (purple).

To further probe the SMM behavior ac magnetic susceptibility measurements were performed (*H*_dc_ = 0, *f* = 1–1000 Hz). A clear frequency-dependent phase shift was observed indicating slow magnetic relaxation and SMM behavior for all complexes. Temperature-dependent magnetic relaxation times were extracted from the data by simultaneously fitting the in-phase (*χ*′) and out-of-phase (*χ*′′) susceptibility to a generalized Debye equation (Fig. 3, S5–S12, ESI†).^15^ Cole–Cole plots (*χ*′ *vs. χ*′′) of the data form semicircles with low eccentricities, indicating that a single relaxation time (*τ*) is associated with each temperature over the frequency ranges studied.1
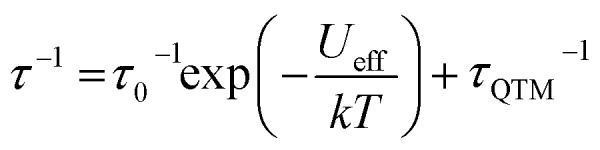

2
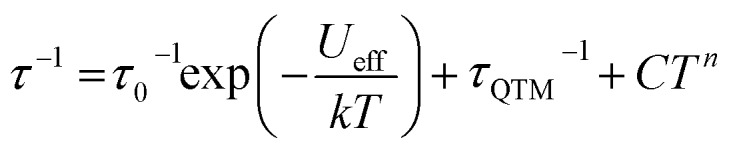



The relaxation behavior of **1–3** can be well-modelled across all temperatures studied with a model containing both Orbach and QTM relaxation terms following eqn (1) (Fig. 3e and S5–S12, ESI†). In the high temperature limit, the thermally-activated over-barrier relaxation (Orbach) mechanism dominates leading to thermal activation barriers to magnetic relaxation of *U*_eff_ = 95.6(9), 102.9(3.1), 107.1(1.3) cm^–1^ for compounds **1–3**, respectively. Attempt times (*τ*_0_) are on the order of 10^–10^ s, consistent with single-molecule relaxation behavior. Below 9 K, the *τ* values begin to show a decreasing dependence on temperature, eventually becoming nearly independent of temperature. In this regime, lack of thermal energy severely limits over-barrier relaxation mechanisms and quantum tunneling of the magnetization (QTM) begins to dominate. QTM processes define the upper limit to the relaxation times and thus explain the lack of remanence in *M vs. H* plots (Table 1, S5–S8 ESI†). Although these two processes capture dominant relaxation mechanisms of **1–3**, an additional term to account for multi-phonon Raman processes was required to fit the relaxation behavior of **4** (eqn (2)). The Raman exponent *n* was restrained to take only integer values. A best fit was obtained with *n* = 5, consistent with a Kramers ion with a multiplet ground state and significant influence from optical phonon modes.^16^,^17^ In this model, **4** had the largest thermal relaxation barrier (*U*_eff_ = 133.6(2.2) cm^–1^). Adding a direct term describing phonon-mediated relaxation within the ground state Kramers doublet did not significantly improve the fit and thus was excluded to limit overparameterization.

**Table 1:** Best-fit parameters of the temperature-dependent relaxation times

	*U*_eff_ (cm^–1^)	*τ*_0_ (s)	*τ*_QTM_ (s)	*C* (K^–*n*^s^–1^)	*n*
**1**	95.6(9)^*b*^	9.2(1.0) × 10^–10^	2.27(4) × 10^–3^	^*a*^	^*a*^
**2**	102.9(3.1)	9.6(2.7) × 10^–10^	2.02(8) × 10^–3^	^*a*^	^*a*^
**3**	107.1(1.3)	6.3(8) × 10^–10^	1.50(2) × 10^–3^	^*a*^	^*a*^
**4**	133.6(2.2)	9.0(1.4) × 10^–10^	1.45(14) × 10^–2^	3.0(8) × 10^–4^	5

^*a*^Indicates an unused fit parameter.

^*b*^Values in parentheses indicate 95% confidence interval uncertainties.

Although **1–4** have similar structures, the larger *U*_eff_ and *τ*_QTM_ of **4** indicate that its higher pseudosymmetry plays an important role in establishing a better isolated and axial ground state Kramers doublet (KD). The lowering of QTM probabilities plays a vital role in the standard design of single-ion magnets,^18^ since desirable temperature-scaling can only be achieved in the Arrhenius regime. When building larger magnetic structures, however, control over QTM is far less important. For our Er–COT-based MLPA building unit, limiting QTM is not nearly as essential as maintaining a well-defined anisotropy, since each additional coupling interaction will enhance the ground state moment and diminish the likelihood of QTM.

Empirically, each variant on the Er–COT unit (**1–4**) provides an example consistent with our MLPA predictions. The discovery that all mononuclear variants of the Er–COT motif show relatively strong over-barrier relaxation provides compelling evidence that the ligation of one COT^2–^ ring to an Er^3+^ center is effective at generating single-ion anisotropy even when the rest of the coordination sphere remains unoptimized. It is interesting to compare these results to Er^3+^ sandwich complexes such as [K(18-crown-6)][Er(COT)_2_]9b,d (*U*_eff_ = 198.8 cm^–1^) [Li(DME)_3_][Er(COT′′)_2_]9*c* (COT′′ = 1,4-bis(trimethylsilyl)COT dianion, DME = dimethoxyethane) (*U*_eff_ = 130 cm^–1^) and [(C_5_H_5_BCH_3_)Ln(COT)]9*e* (*U*_eff_ = 300 cm^–1^) which have rigorously demonstrated the effectiveness of Er^3+^ ions when placed in a more optimal coordination sphere. Clearly, bis-COT or mixed ring structures are effective for Er^3+^, so the retention of SMM behavior with a single COT ring could be taken as a given. Lanthanide anisotropy, however, is almost wholly determined by the immediate coordination sphere and even subtle changes can cause catastrophic losses in magnetic anisotropy.4c,9e,^19^ Importantly, **1–4** are able to retain anisotropy despite deliberate de-optimization of their coordination sphere.

### Computational studies

**Fig. 4:**
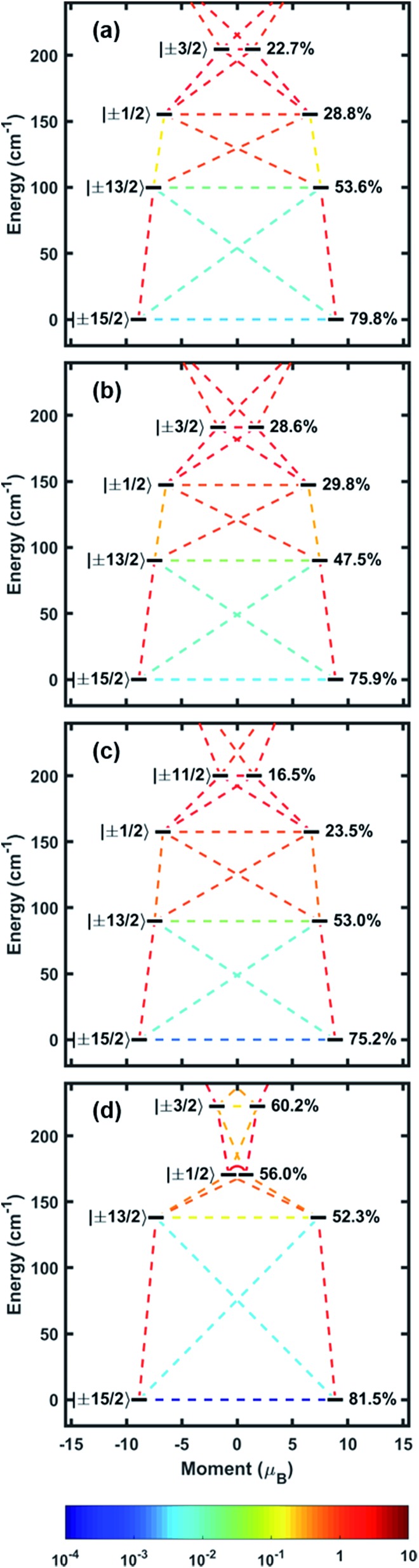
Calculated energy spectra of the four lowest-lying Kramers states as a function of their magnetic moments for (a) **1**, (b) **2**, (c) **3** and (d) **4**. The states are labelled by their largest contributing M_*J*_ component (left) and the percentage of that component (right). Colored lines connecting the states represent matrix elements of the transition magnetic moment ((|*μ*_*x*_| + |*μ*_*y*_| + |*μ*_*z*_|)/3) calculated between them.

With experimental evidence in hand, we sought further insight into the exact nature of the electronic states leading to our robust SMM behavior. Electronic structures of **1–4** were modelled with MOLCAS 8.0 using complete-active space self-consistent field (CASSCF) methods.^20^ Input atom coordinates for these calculations were taken from X-ray data and used without further geometry optimization. The orientation of the main magnetic axes and the *g*-tensors belonging to the ground state doublets have been calculated (Fig. 2, Table 2). These calculations confirm that the Er–COT unit not only enforces uniaxial anisotropy, but also enforces the orientation axis of that anisotropy. This orientation is fixed to the COT^2–^ normal vector with a maximum deviation of only 6.25°. These results indicate that the Er–COT unit can achieve maximal anisotropy contributions from each ion given a judicious choice of bridging ligand to force COT^2–^ units to be parallel. Not surprisingly given its symmetric coordination mode, the bistable ground state of compound **4** possess the smallest transverse *g* component and is consequently the most axially magnetic. As discussed elsewhere,^21^ transverse magnetic moment matrix elements calculated between states are roughly proportional to their respective transition rates. The calculated energies of the four lowest-lying Kramers states along with the transverse magnetic moment matrix elements connecting these states indicate that the most probable relaxation pathways overwhelmingly involve the ground and first excited doublets, KD_2–1_ (Fig. 4, Table 2, Tables S3–S6†). The splitting between these two states in each compound matches closely the effective barriers (*U*_eff_) that were extracted from ac susceptibility data. This finding supports the rationale that at high temperature **1–4** relax largely *via* an over-barrier (Orbach) mechanism involving the first excited KD. The purity of the spin–orbit states is relatively low compared to synthetically-optimized SMMs. Consequently, matrix elements between them are found to be non-negligible and QTM is expected to play an important role toward relaxation in zero field. These results agree with the lack of hysteresis in the *M vs. H* plots. Since QTM only has a strong effect on zero-dimensional magnetism (*i.e.* SMM), it is important to reiterate that state-purity or its manifestation as magnetic hysteresis is not a requirement for a successful MLPA unit.

**Table 2:** Selected magnetic parameters of the ground state Kramers doublets

	*g*_*x*_	*g*_*y*_	*g*_*z*_	*θ* (°)	ΔKD_2–1_ (cm^–1^)	*U*_eff_ (cm^–1^)
**1**	0.007	0.011	17.820	1.49	99.8	95.6(9)
**2**	0.009	0.015	17.747	2.61	90.1	102.9(3.1)
**3**	0.002	0.005	17.707	6.25	89.8	107.1(1.3)
**4**	0.000	0.001	17.722	0.74	138.0	133.6(2.2)

## Conclusions

We have systematically tested a design principle for the rational expansion of anisotropic single-ion magnets to clusters and higher dimensionality structures. This principle of Metal–Ligand Pair Anisotropy (MLPA) fixes the anisotropy axis of a coordinatively reactive lanthanide relative to a strong magnetic directing ligand. To demonstrate the feasibility of MLPA, variants on the Er–COT metal ligand pair have been synthesized and magnetically analyzed. Although each molecule represents a different distortion on the Er–COT structure, they all display SMM behavior with a largely M_*J*_ = ±15/2 ground KD that is normal to the COT ring. Future work will focus on understanding how magnetic coupling perturbs the Er–COT anisotropy and the extent to which magnetically-coupled materials can be synthesized with rationally designed anisotropy.

## Conflicts of interest

There are no conflicts to declare.

## Supplementary Material

Supplementary information

Crystal structure data
