# Supplementary material for: Metal–ligand pair anisotropy in a series of mononuclear Er–COT complexes
Source: Chem Sci. 2018 Jul 31;9(36):7204–9. doi: 10.1039/c8sc01361f (PMC6335627; doi:10.1039/c8sc01361f)
Supplement: Supplementary file 1 [file SC-009-C8SC01361F-s001.pdf]

Electronic Supplementary Material (ESI) for Chemical Science.
This journal is © The Royal Society of Chemistry 2018

Contents

1. **General considerations and synthesis of compounds** 1
    - **1.1.** (η^8-cyclooctatetraenyl)-bis(tetrahydrofuran)-erbium-iodide (**1**) synthesis
    - **1.2.** (η^8-cyclooctatetraenyl)-bis(pyridine)-erbium-iodide (**2**) synthesis
    - **1.3.** (η^8-cyclooctatetraenyl)-bis(acetonitrile)-erbium-iodide (**3**) synthesis
    - **1.4.** (η^8-cyclooctatetraenyl)-tris(3,5-dimethyl-1-pyrazolyl)borate erbium **(4)** synthesis
2. **Crystallographic information** 3
3. **Magnetic analyses** 5
4. **Computational information** 9
5. **References** 14

1. General considerations and synthesis of compounds:

All manipulations were carried out under an atmosphere of dinitrogen using standard Schlenk line and glovebox techniques. THF and diethyl ether were dried on an activated alumina column and stored on 3 Å molecular sieves for two days before use. Anhydrous pyridine was purchased from Sigma and stored on 3 Å molecular sieves for one week before use. ErI_3 powder and COT were purchased from Aldrich and used as received. Dipotassium cyclooctatetraenide was prepared via a previously reported method.[1] Elemental analyses were conducted by Midwest Microlab, Indianapolis, IN.

1.1. (η^8-cyclooctatetraenyl)-bis(tetrahydrofuran)-erbium-iodide (**1**) synthesis:

Cold K_2COT (30.25 mg, 0.166 mmol, 1 equiv) in 6 mL cold THF (-45 °C) was added to a stirring cold (-45 °C) mixture of ErI_3 (100 mg, 0.182 mmol, 1.1 equiv) in 8 mL THF. The mixture was allowed to warm to room temperature and stir for 16 hours. The resultant bright pink suspension was centrifuged (3200 rpm, 20 minutes) and the supernatant separated and dried *in vacuo*. The dried solids were suspended in 10 mL THF before heating to 60 °C in a sealed scintillation vial to form a clear pink solution. Diethyl ether was diffused into this solution, and the solution was afterward stored at -45 °C to yield pink blocks of **1** (58.4 mg, 65.2%). CHN analysis (calc., found) for $C_{16}H_{16}IErO_2$: C (35.42 ,34.94); H (4.46, 4.22); N (0.00, 0.00).

1.2. (η^8-cyclooctatetraenyl)-bis(pyridine)-erbium-iodide (**2**) synthesis

A cold solution of K_2COT (26.34 mg, 0.144 mmol, 0.9 equiv) in 6 mL pyridine was added to a stirring cold mixture of ErI_3 (88.0 mg, 0.161 mmol, 1 equiv) in 8 mL pyridine at ambient temperature. After stirring for 2 hours, the yellow mixture was centrifuged (3200 rpm, 20 minutes) and the supernatant separated and dried *in vacuo*. The dried yellow solid was stirred in toluene at 50 °C in a clear centrifuge vial and filtered. Hexane was diffused into this solution to yield dark orange plates of **2** (51.3 mg, 64.0%). CHN analysis (calc., found) for $C_{18}H_{18}IErN_2$: C (38.85, 38.38); H (3.26, 3.25); N (5.03, 4.95)

1.3. (η^8-cyclooctatetraenyl)-bis(acetonitrile)-erbium-iodide (**3**) synthesis:

A solution of η^8-(cyclooctatetraenyl)-bis(tetrahydrofuran)-erbium-iodide (66.5 mg, 0.182 mmol, 1 equiv) was stirred in 10 mL acetonitrile at 50 °C for 24 hours, during which it slowly changed from a suspension to an orange solution. The solution was then centrifuged, and the resultant supernatant was slowly dried *in vacuo* until about 1 mL solution remained and stored at -45 °C to form x-ray quality crystals of **3** (39.3 mg, 66.7 %). CHN analysis (calc., found) for $C_{12}H_{14}IErN_2$: C (30.00, 29.87; H (2.94, 3.04); N (5.83, 5.61)

1.4. (η^8-cyclooctatetraenyl)-tris(3,5-dimethyl-1-pyrazolyl)borate-erbium (**4**) synthesis

A solution of potassium tris(3,5-dimethyl-1-pyrazolyl)borate (60.8 mg, 0.188 mmol) in THF was added to a mixture of η^8-(cyclooctatetraenyl)-bis(tetrahydrofuran)-erbium-iodide (102.1 mg, 0.188 mmol, 1 equiv) in THF and stirred for 24 h. The resultant light pink solution was centrifuged (3200 rpm, 20 minutes) and the supernatant separated and concentrated *in vacuo*. The concentrated solution was cooled to -45 °C to form bright pink crystals of **4** (37.5 mg, 61.8%). CHN analysis (calc., found) for $C_{23}H_{31}IErN_6$: C (48.50, 47.33); H (5.49, 5.23); N (14.75, 14.26)

2. Crystallographic Information:

Single crystal X-ray data for **1** was collected at 230 K and **2**, **3**, and **4** were collected at 200 K on a Bruker κ Diffractometer with a Mo Kα radiation source and an Apex II Area Detector. The structure was solved using direct methods via the SHELXT2[2] routine and refined using full-matrix least-squares procedures with the SHELXL routine. **Olex²** [3] was used as a graphical front-end. Hydrogens were modelled using a riding model for all positions except the borate hydrogen in **4**.

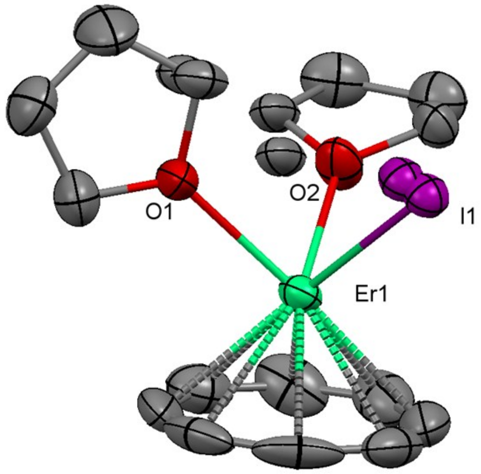

Figure S1. Solid-state ORTEP for **1**. The thermal ellipsoids are represented at 50% probability level. Hydrogen atoms have been omitted for clarity. Selected distances: Er1-COT (Centroid), 1.77135(1) Å; Er1-I1, 3.0648 (2 Å); Er1-O1, 2.35372(2) Å; Er1-O2, 2.47498(2) Å

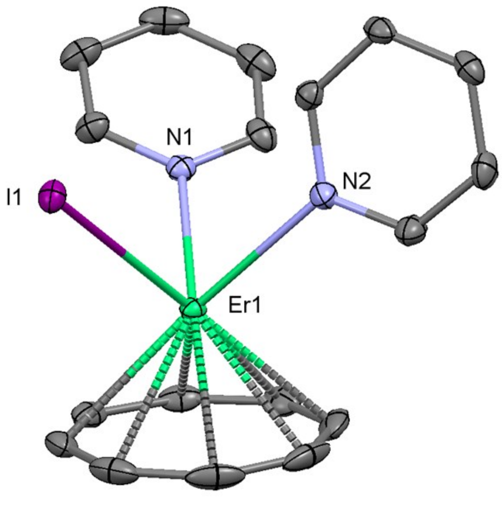

Figure S2. Solid-state ORTEP for **2**. The thermal ellipsoids are represented at 50% probability level. Hydrogen atoms have been omitted for clarity. Selected distances: Er1-COT (Centroid), 1.77034(9) Å; Er1-I1, 3.02712(18) Å; Er1-N1, 2.46446(17) Å; Er1-N2, 2.50914(2) Å

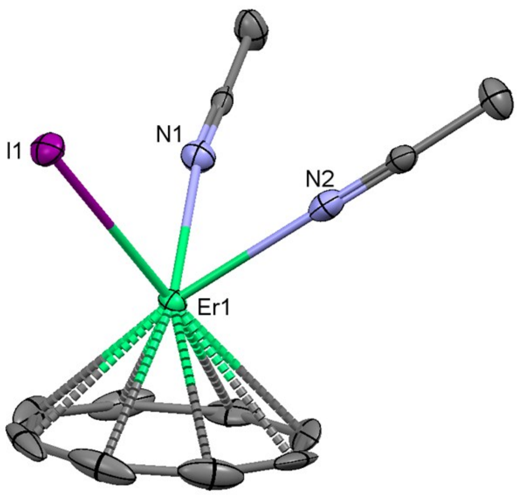

Figure S3. Solid-state ORTEP for **3**. The thermal ellipsoids are represented at 50% probability level. Hydrogen atoms have been omitted for clarity. Selected distances: Er1-COT (Centroid), 1.74885(1) Å; Er1-I1, 3.0024(2) Å; Er1-N1, 2.43472(2) Å; Er1-N2, 2.4304(2) Å;

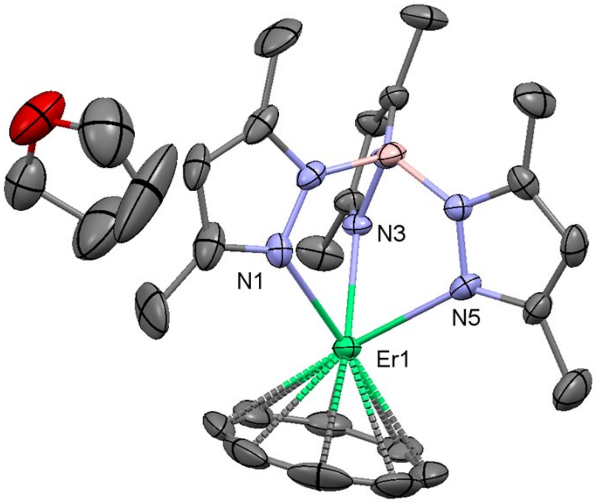

Figure S4. Solid-state ORTEP for **4**. The thermal ellipsoids are represented at 50% probability level. Hydrogen atoms have been omitted for clarity. Selected distances: Er1-COT (Centroid), 1.8359(5) Å; Er1-N1, 2.483(4) Å; Er1-N3, 2.489(4) Å; Er1-N5, 2.478(4) Å

3. Magnetic analyses

3.1. (η^8-cyclooctatetraenyl)-bis(tetrahydrofuran)-erbium-iodide (**1**)

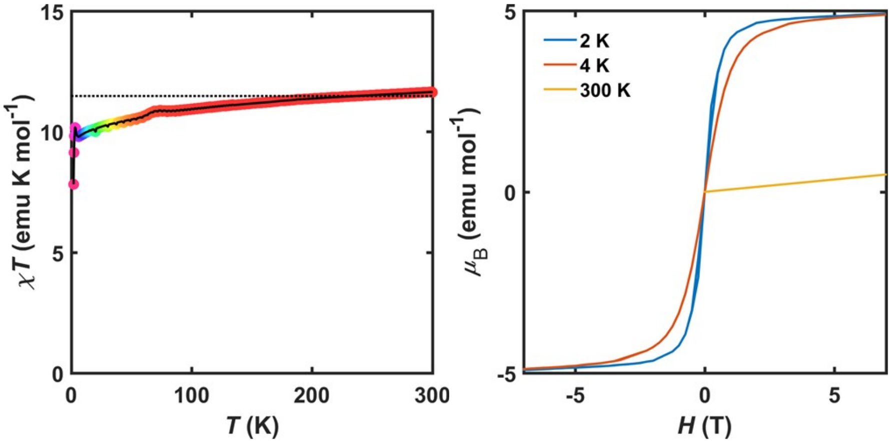

Figure S5. Static magnetic properties of **1**. Shown are the thermal susceptibility (left) measured at 1000 Oe with dotted line showing theoretical χ_T value and isothermal magnetization (right).

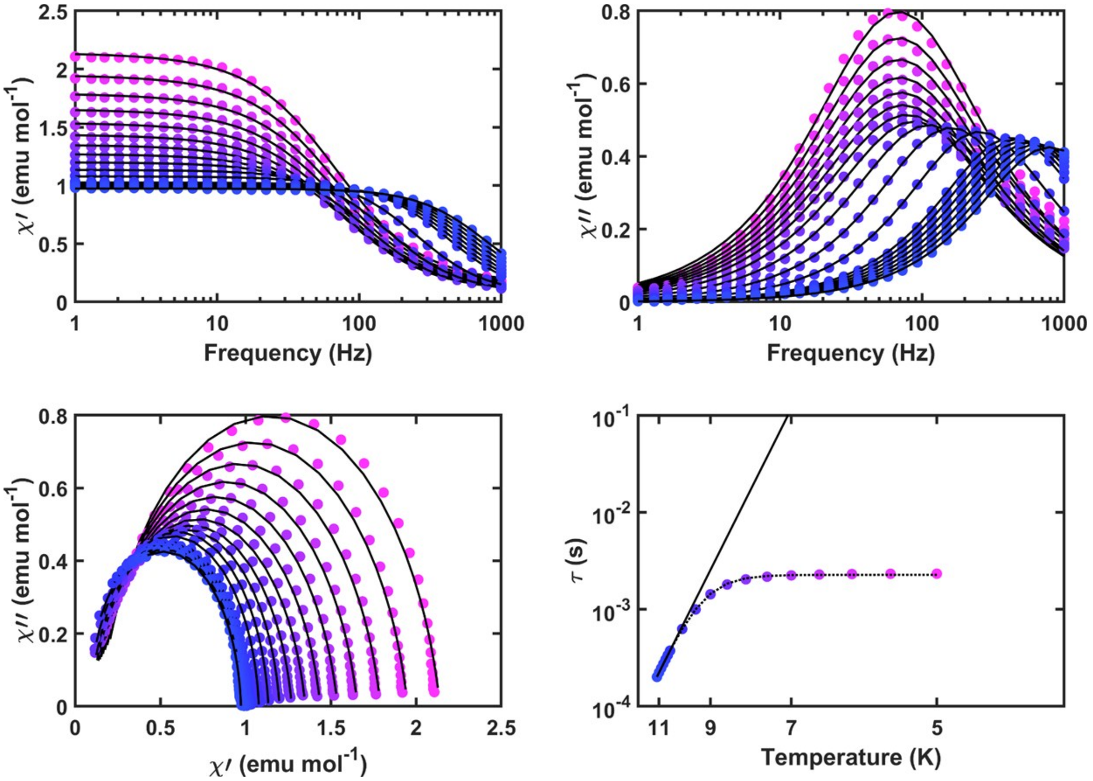

Figure S6. Dynamic magnetic properties of **1.** Clockwise from top-left: in-phase susceptibility *vs.* frequency, out-susceptibility *vs.* frequency, Cole-Cole plot, Arrhenius plot. Arrhenius plot: solid line is the Arrhenius fit at high temperature (U_{eff} = 83.8(1.5) cm^{-1}; τ_0 = 3.9(6)x10^{-9} s) and the dashed line is the fit to eqn 1 (U_{eff} = 95.6(9) cm^{-1}; τ_0 = 9.2(1.0)x10^{-10} s; τ_{QTM} = 0.00227(4) s).

3.2. (η^8-cyclooctatetraenyl)-bis(pyridine)-erbium-iodide (**2**)

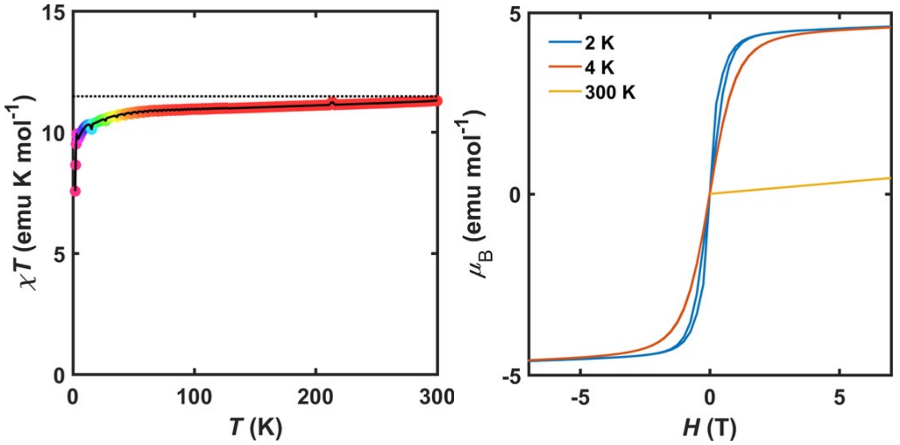

Figure S7. Static magnetic properties of **2**. Shown are the thermal susceptibility (left) measured at 1000 Oe with dotted line showing theoretical χ_T value and isothermal magnetization (right).

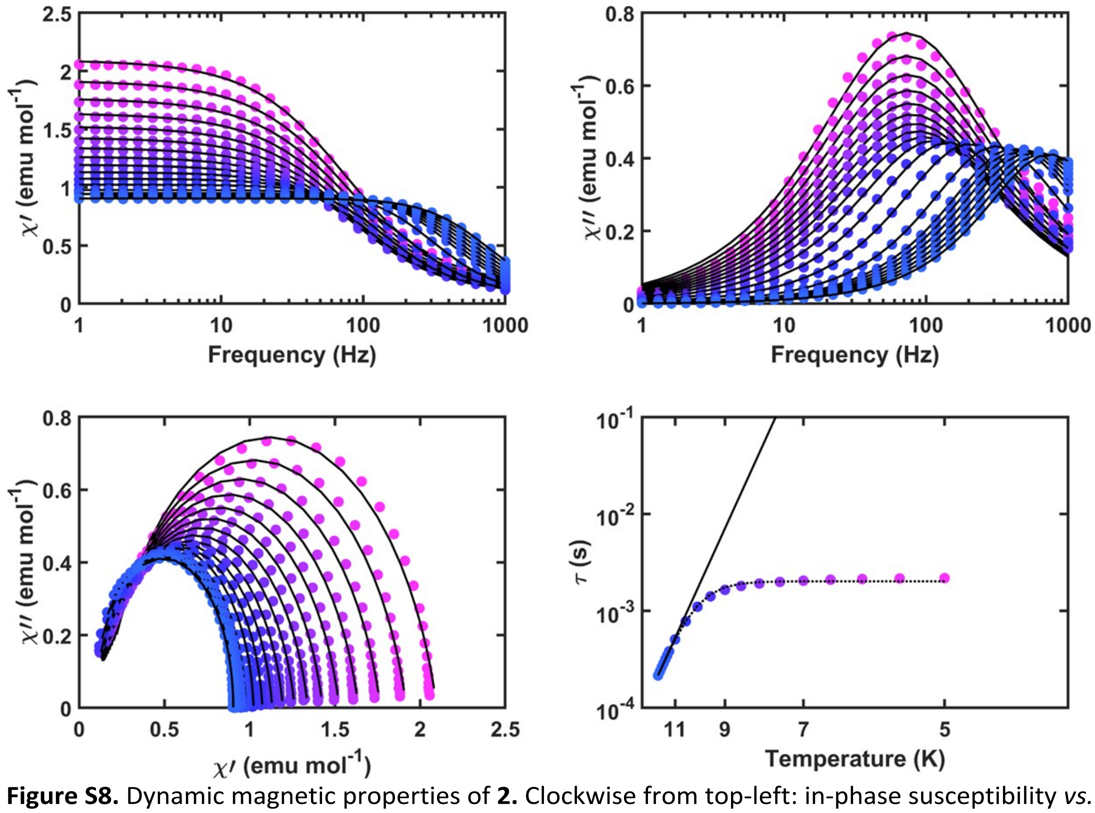

Figure S8. Dynamic magnetic properties of **2.** Clockwise from top-left: in-phase susceptibility *vs.* frequency, out-susceptibility *vs.* frequency, Cole-Cole plot, Arrhenius plot. Arrhenius plot: solid line is the Arrhenius fit at high temperature (U_{eff} = 89.6(1.5) cm^{-1}; τ_0 = 4.3(6)x10^{-9} s) and the dashed line is the fit to eqn 1 (U_{eff} = 102.9(3.1) cm^{-1}; τ_0 = 9.6(2.7)x10^{-10} s; τ_{QTM} = 0.00202(8) s).

3.3. (η^8-cyclooctatetraenyl)-bis(acetonitrile)-erbium-iodide (**3**)

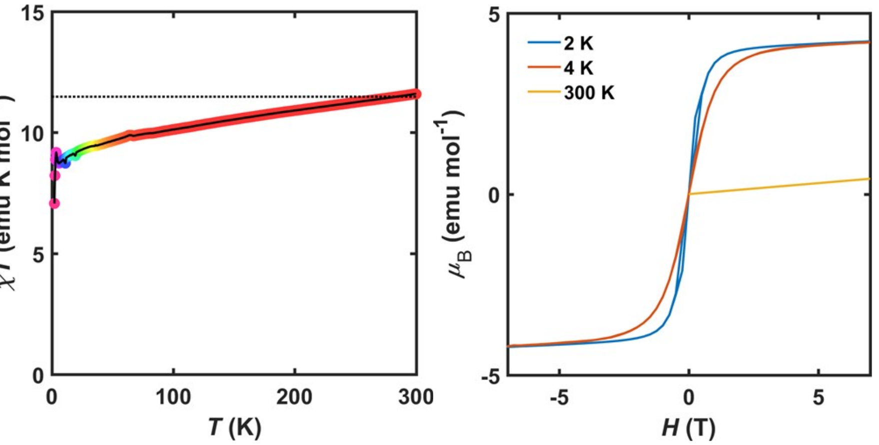

Figure S9. Static magnetic properties of **3**. Shown are the thermal susceptibility (left) measured at 1000 Oe with dotted line showing theoretical χ_T value and isothermal magnetization (right).

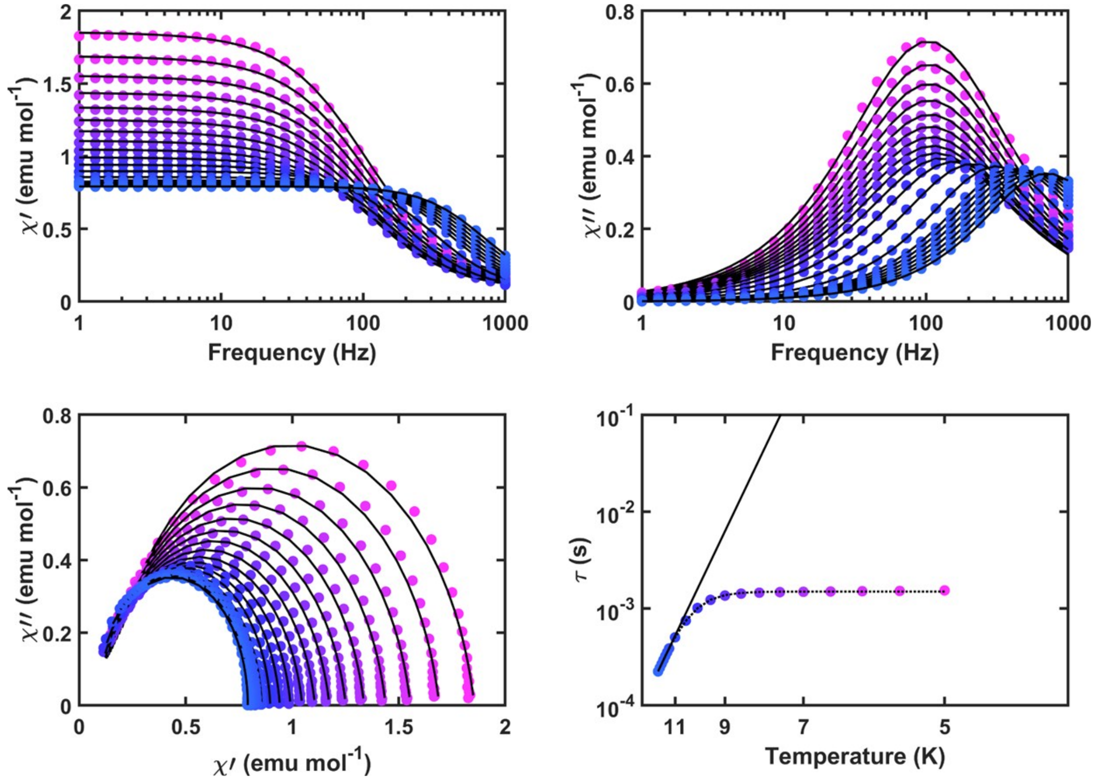

Figure S10. Dynamic magnetic properties of **3.** Clockwise from top-left: in-phase susceptibility *vs.* frequency, out-susceptibility *vs.* frequency, Cole-Cole plot, Arrhenius plot. Arrhenius plot: solid line is the Arrhenius fit at high temperature (U_{eff} = 85.5(1.5) cm^{-1}; τ_0 = 7.3(1.0)x10^{-9} s) and the dashed line is the fit to eqn 1 (U_{eff} = 107.1(1.3) cm^{-1}; τ_0 = 6.3(8)x10^{-10} s; τ_{QTM} = 0.00150(2) s).

3.4. (η[8]-cyclooctatetraenyl)-tris(3,5-dimethyl-1-pyrazolyl)borate-erbium (**4**)

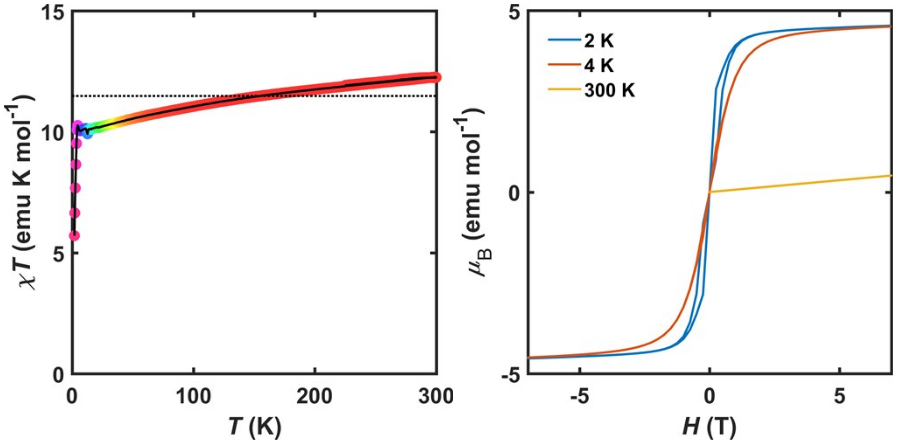

Figure S11. Static magnetic properties of **4**. Shown are the thermal susceptibility (left) measured at 1000 Oe with dotted line showing theoretical χ_T value and isothermal magnetization (right).

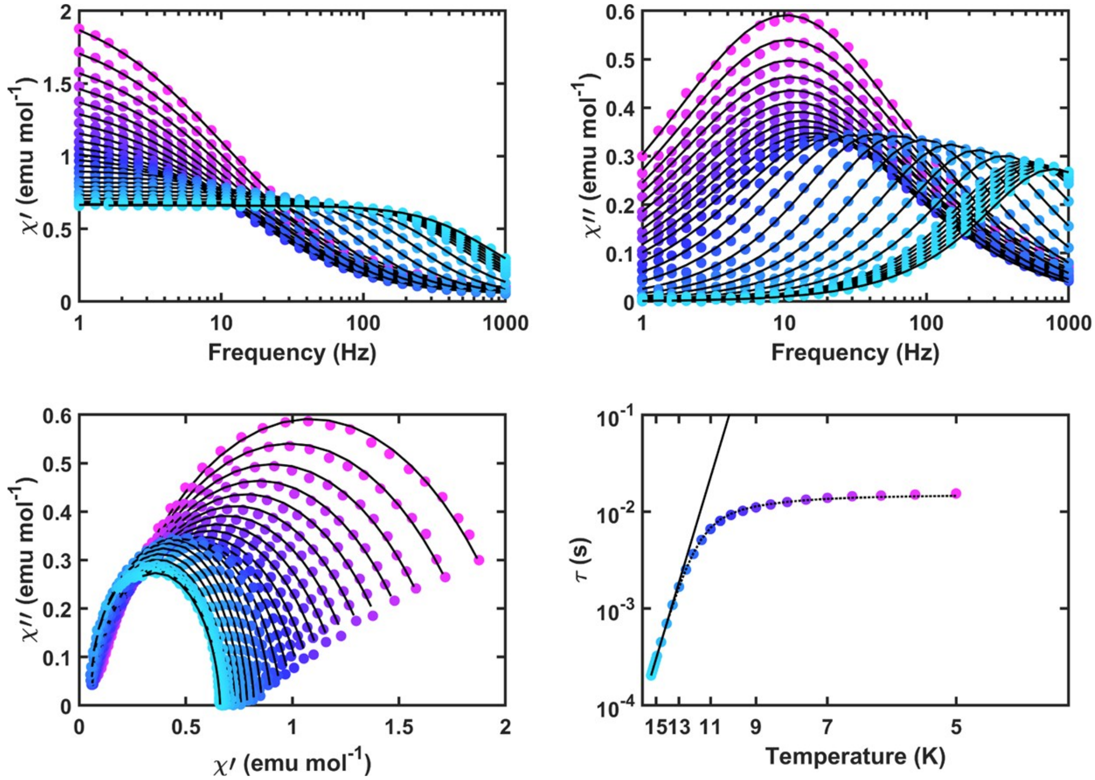

Figure S12. Dynamic magnetic properties of **4.** Clockwise from top-left: in-phase susceptibility *vs.* frequency, out-susceptibility *vs.* frequency, Cole-Cole plot, Arrhenius plot. Arrhenius plot: solid line is the Arrhenius fit at high temperature (U_{eff} = 123.9(1.9) cm^{-1}; τ_0 = 2.1(3)x10^{-9} s) and the dashed line is the fit to eqn 2 (U_{eff} = 133.6(2.2) cm^{-1}; τ_0 = 9.0(1.4)x10^{-10} s; τ_{QTM} = 0.0145(14) s; C = 3.0(8)x10^{-4}; n = 5).

4. Computational information

All calculations were performed using the SEWARD/RASSCF/RASSI/SINGLE_ANISO modules of MOLCAS 8.0. Input atom coordinates were taken from crystallographic data and used without further geometry optimization. Compound **1** displayed disorder over the iodide and THF ligands – the higher occupancy positions were used for the calculations. The outer sphere THF in the crystal structure of **4** was not included in the input atom coordinates. Basis sets of the ANO-RCC type were utilized and the specific functions for each atom-type is shown below (Table S1). A CAS(11,7) active-space – corresponding to 11 electrons in 7 *f*-orbitals – was used for the self-consistent field procedure. In this space we included 35 CI roots of spin multiplicity 4 and 112 CI roots of spin multiplicity of 2. Spin-orbit coupling was accounted for using the RASSI module (all RASSCF states were used), the output of which was directed to SINGLE_ANISO to extract all relevant magnetic information.

Table S1. Utilized basis set.

**Er.ANO-RCC...8s7p5d3f2g1h**
**B.ANO-RCC...3s2p1d**
**C.ANO-RCC...3s2pd1**
**H.ANO-RCC...2s**
**I.ANO-RCC...6s5p3d1f**
**N.ANO-RCC...3s2p1d**
**O.ANO-RCC...3s2p1d**

Table S2. J = 15/2 energy spectra.

	**1**		**2**		**3**		**4**	
**KD**	μ_B	E_{KD} (cm^{-1})	μ_B	E_{KD} (cm^{-1})	μ_B	E_{KD} (cm^{-1})	μ_B	E_{KD} (cm^{-1})
**1**	8.910011588	0	8.873484578	0	8.853460946	0	8.861021448	0
**2**	7.548457229	99.817	7.454311347	90.087	7.45654627	89.839	7.333484582	138.039
**3**	6.570541319	155.267	6.425876122	147.294	6.706785948	157.455	0.768839679	170.515
**4**	1.41264312	204.545	1.721778778	190.968	1.522234022	200.077	1.82656634	222.461
**5**	5.57790397	258.237	5.7072727	253.529	5.275771776	279.282	4.716428086	273.219
**6**	5.561225175	313.87	4.540468799	307.545	3.967697913	328.589	4.882327234	334.221
**7**	5.436224585	368.626	4.843518644	352.003	5.50248555	365.787	5.305482768	384.054
**8**	7.360863602	404.286	7.080765745	380.135	6.21712003	383.378	6.368779964	394.545

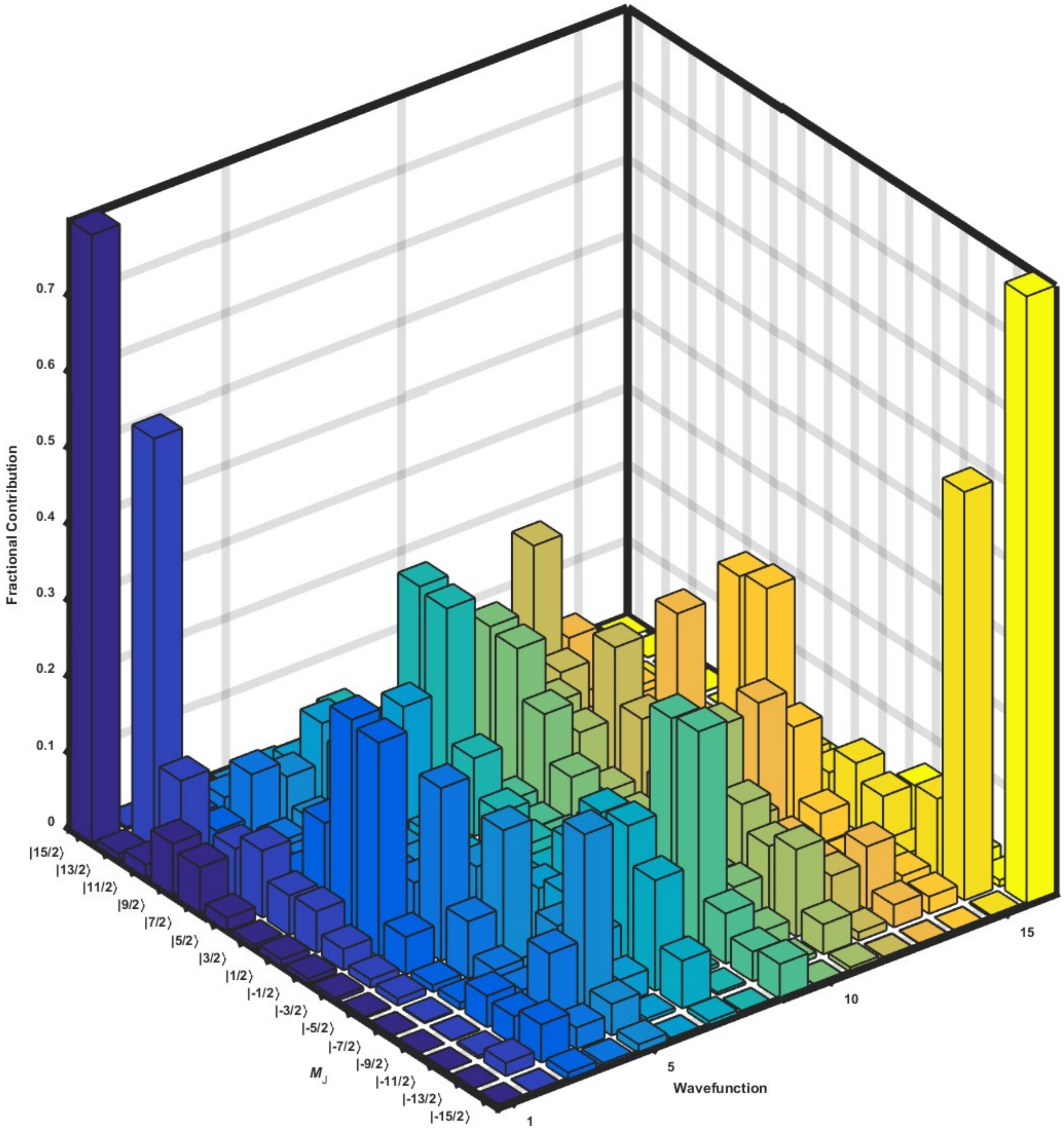

Figure S13. RASSI wavefunction components calculated for **1**. Bar heights represent normalized wavefunction decompositions of the lowest 16 RASSI states (8 Kramers doublets) in terms of wavefunctions with definite projection of the total moment $|M_J\rangle$.

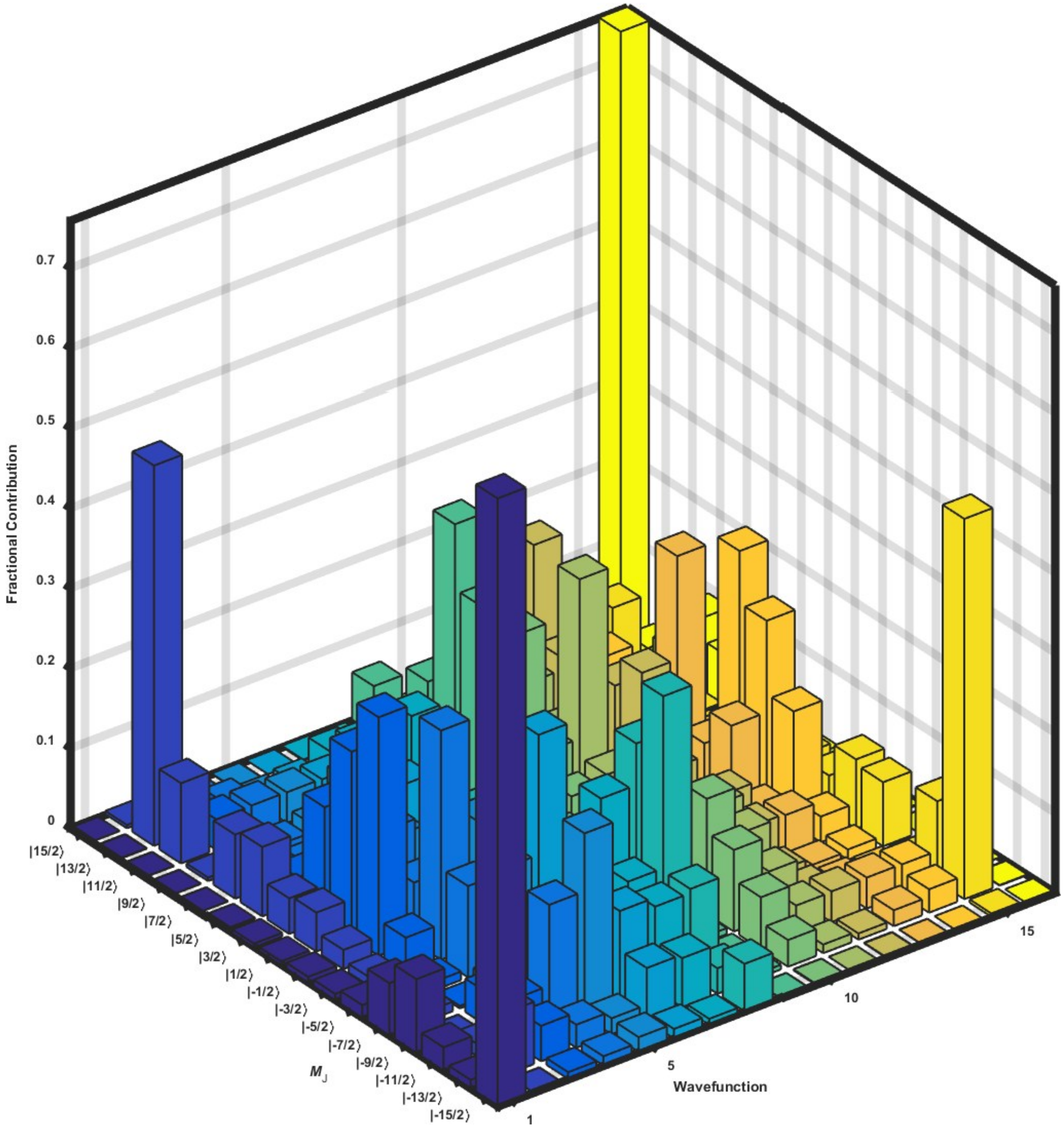

Figure S14. RASSI wavefunction components calculated for **2**. Bar heights represent normalized wavefunction decompositions of the lowest 16 RASSI states (8 Kramers doublets) in terms of wavefunctions with definite projection of the total moment $|M_J\rangle$.

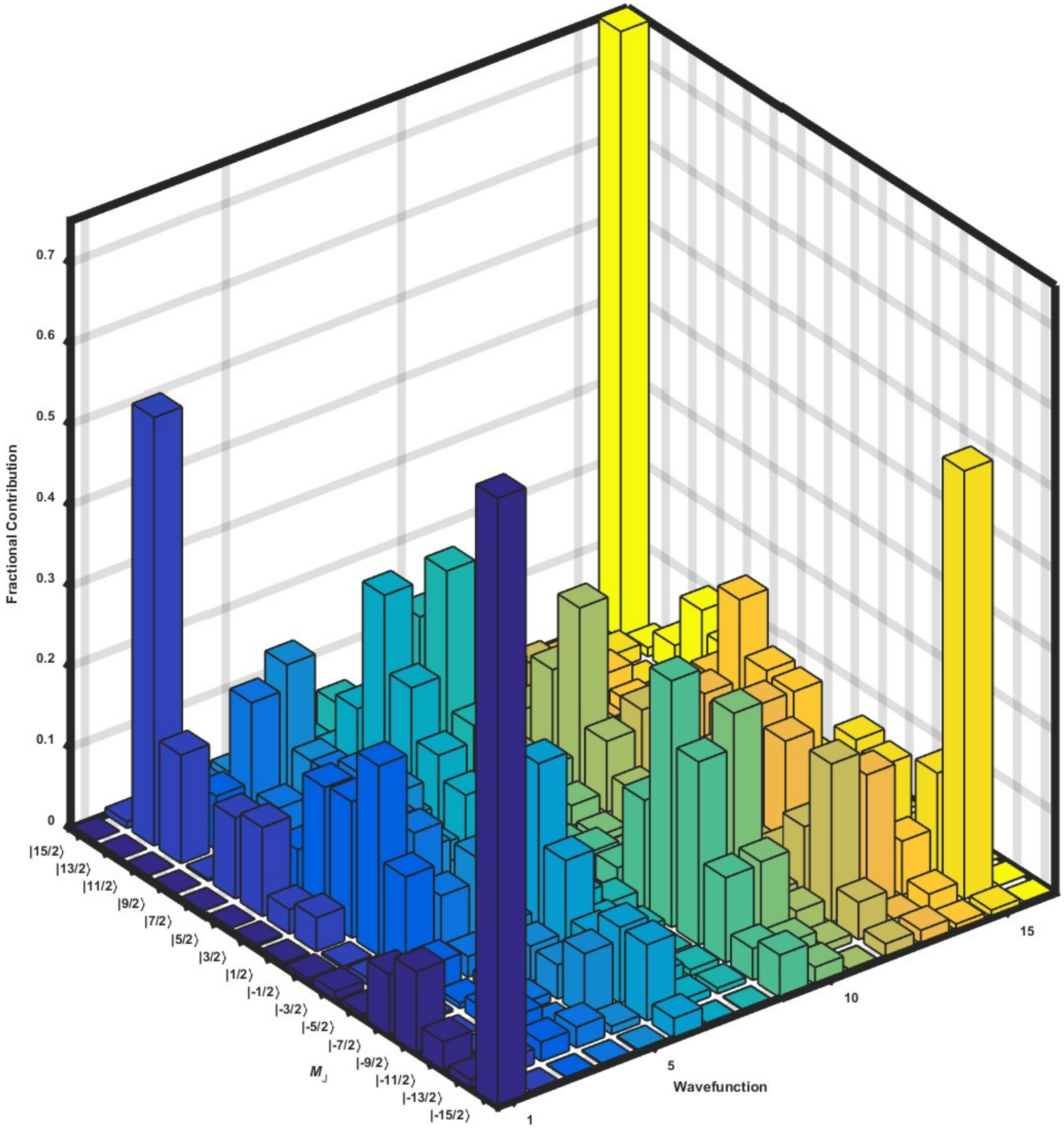

Figure S15. RASSI wavefunction components calculated for **3**. Bar heights represent normalized wavefunction decompositions of the lowest 16 RASSI states (8 Kramers doublets) in terms of wavefunctions with definite projection of the total moment $|M_J\rangle$.

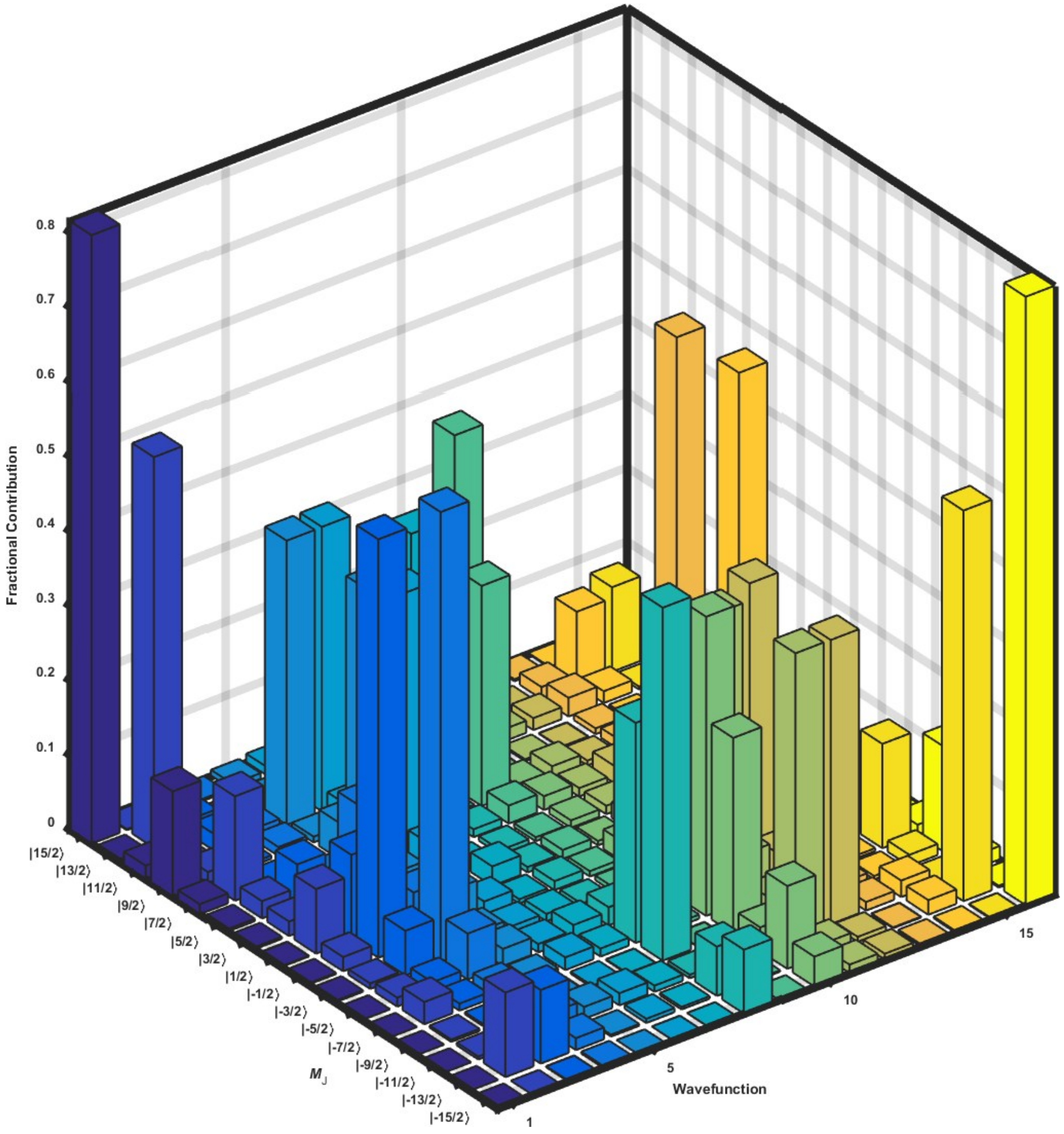

Figure S16. RASSI wavefunction components calculated for **4**. Bar heights represent normalized wavefunction decompositions of the lowest 16 RASSI states (8 Kramers doublets) in terms of wavefunctions with definite projection of the total moment $|M_J\rangle$.

Table S3. *Selected average magnetic moment matrix elements between the J = 15/2 multiplets of **1**.*

Multiplet (i)	$\langle i_\uparrow \vert \hat{M}_{avg} \vert i_\downarrow \rangle$	$\langle i_\uparrow \vert \hat{M}_{avg} \vert i_\uparrow + 1 \rangle$	$\langle i_\uparrow \vert \hat{M}_{avg} \vert i_\downarrow + 1 \rangle$
**1**	0.297846714387E-02	0.154950309040E+01	0.925826562725E-02
**2**	0.206655819195E-01	0.192868958058E+00	0.949713259036E+00
**3**	0.109255802511E+01	0.222303027203E+01	0.170132450799E+01
**4**	0.198071836348E+01	0.119733714058E+01	0.173999058754E+01
**5**	0.421981486320E+00	0.710533669619E+00	0.193559044539E+01
**6**	0.612739473858E+00	0.811143265161E+00	0.175758897611E+01
**7**	0.597064201612E+00	0.373888330460E+00	0.196889694935E+01
**8**	0.256780142976E+00		

Table S4. *Selected average magnetic moment matrix elements between the J = 15/2 multiplets of **2**.*

Multiplet (i)	$\langle i_\uparrow \vert \hat{M}_{avg} \vert i_\downarrow \rangle$	$\langle i_\uparrow \vert \hat{M}_{avg} \vert i_\uparrow + 1 \rangle$	$\langle i_\uparrow \vert \hat{M}_{avg} \vert i_\downarrow + 1 \rangle$
**1**	0.410015169230E-02	0.157299357721E+01	0.111945873915E-01
**2**	0.345565703589E-01	0.332270991731E+00	0.110355993346E+01
**3**	0.921852176295E+00	0.200434399632E+01	0.188766383514E+01
**4**	0.192802065215E+01	0.126657179218E+01	0.161643207820E+01
**5**	0.426866235884E+00	0.199850440194E+01	0.818360422228E+00
**6**	0.901430727044E+00	0.192739153139E+01	0.853748288688E+00
**7**	0.776795369566E+00	0.189480834824E+01	0.548440158679E+00
**8**	0.320809641204E+00		

Table S5. *Selected average magnetic moment matrix elements between the J = 15/2 multiplets of **3**.*

Multiplet (i)	$\langle i_{\uparrow}\|\hat{M}_{avg}\|i_{\downarrow}\rangle$	$\langle i_{\uparrow}\|\hat{M}_{avg}\|i_{\uparrow}+1\rangle$	$\langle i_{\uparrow}\|\hat{M}_{avg}\|i_{\downarrow}+1\rangle$
**1**	0.119496821393E-02	0.158396090236E+01	0.891980630891E-02
**2**	0.319074138924E-01	0.630738715975E+00	0.937948870168E+00
**3**	0.973112984563E+00	0.184428203337E+01	0.184975530051E+01
**4**	0.250138503200E+01	0.157519334911E+01	0.118167961183E+01
**5**	0.845146992458E+00	0.108834698069E+01	0.183177048282E+01
**6**	0.104626013324E+01	0.769032140609E+00	0.200006846417E+01
**7**	0.611313025991E+00	0.234514765358E+01	0.448790759956E+00
**8**	0.285132142267E+00		

Table S6. *Selected average magnetic moment matrix elements between the J = 15/2 multiplets of **4**.*

Multiplet (i)	$\langle i_{\uparrow}\|\hat{M}_{avg}\|i_{\downarrow}\rangle$	$\langle i_{\uparrow}\|\hat{M}_{avg}\|i_{\uparrow}+1\rangle$	$\langle i_{\uparrow}\|\hat{M}_{avg}\|i_{\downarrow}+1\rangle$
**1**	0.121926287772E-03	0.157905696475E+01	0.460757876574E-02
**2**	0.133515743420E+00	0.506985935563E+00	0.700940590789E+00
**3**	0.308347320469E+01	0.314198126600E+01	0.328428407865E+00
**4**	0.208328933119E+00	0.223877426628E+01	0.362952015262E+00
**5**	0.166492685012E+00	0.740357780011E+00	0.156226075210E+00
**6**	0.199218738447E+00	0.221237731079E+01	0.146097517375E+00
**7**	0.202959608568E+00	0.239867339989E+01	0.159388182907E+00
**8**	0.962418010518E-01		

5. References

1. Meihaus, K. R.; Long, J. R., *J. Am. Chem. Soc.* **2013,** *135* (47), 17952-17957.
2. Sheldrick, G. M., *Acta Crystallogr. C Struct. Chem.* **2015,** *71* (Pt 1), 3-8.
3. Dolomanov, O. V.; Bourhis, L. J.; Gildea, R. J.; Howard, J. A. K.; Puschmann, H., *J. Appl. Crystallogr.* **2009,** *42*, 339-341.
